# Vulnerabilities, extreme weather and temporal tensions as experienced by construction workers in the Swedish construction sector

**DOI:** 10.1371/journal.pone.0345707

**Published:** 2026-04-24

**Authors:** Bo Nilsson, Anna Sofia Lundgren, Jenny Lönnroth

**Affiliations:** Department of Culture and Media Studies, Umeå University, Umeå, Sweden; Federal University Otuoke, NIGERIA

## Abstract

**Aim:**

Climate change poses increasing risks to outdoor occupations, including construction work. This study explores how vulnerability is constructed in narratives of manual labour within the Swedish construction sector, particularly under extreme weather conditions.

**Methods:**

Drawing on 16 qualitative interviews with Swedish construction workers, the study adopts a social constructionist lens to explore how vulnerability is shaped and experienced.

**Findings:**

The findings identify multiple, intersecting forms of vulnerability—bodily, hierarchical, material, social, and market-driven—exacerbated by climate-related challenges such as high temperatures, heavy rainfall, and strong winds. Crucially, the analysis highlights how extreme weather disrupts temporal rhythms, widening the gap between scheduled work plans and the actual time needed to complete tasks.

**Conclusion:**

The paper concludes that vulnerability arises not only from direct exposure to adverse weather, but also through indirect social, material, and temporal dynamics inherent in the construction sector and exacerbated by climate change.

## Introduction

According to Swedish members of parliament, the climate crisis is acute: ‘Around the world, people and animals are suffering from extreme droughts, floods, and water shortages’ [1]. The European Union likewise warns that Europe will be increasingly afflicted by heatwaves, droughts, intense rainfall and floods. In the summer of 2024, Poland, Austria, and the Czech Republic experienced severe flooding, as did Sweden. It has been established that the increasing levels of rainfall and rising temperatures already being seen in Sweden are an effect of climate change [2]. Significant economic impacts are also attributed to climate change [3].

Climate change and altered weather conditions affect many occupational groups in Sweden, particularly those who work outdoors, such as industrial workers, farmers, and road workers, for whom the changing climate sometimes makes it impossible to carry out aspects of their work [4]. Workers in the construction industry are especially exposed, with heatwaves during the summer months being a recurring problem, while heavy rainfall, strong winds, and challenging ice and snow conditions also create difficult working conditions [5,6]. This has led to an increase in work-related risks, especially in manual outdoor occupations. For example, between 1992 and 2016, 36% of all occupational heat-related deaths in the United States were among construction workers [7].

A defining trend in Sweden’s labour market in recent years has been its gradual deregulation, which is marked by reduced government oversight and a shift towards market-driven principles characteristic of capitalist production. While some argue that increased competition has benefited various industries [8], others view this development more critically—particularly within the construction sector, which has seen a rise in unfair competition, the influence of international organised crime, and the growth of unscrupulous companies [9]. Deregulation has also made economic growth, profit margins, and time efficiency central concerns. The latter is particularly crucial in the construction industry, where shorter construction times are a key and much-sought-after objective, and is often cited as a reason why many construction workers regularly report feeling stressed [10,11].

Construction stress is linked to workplace accidents, and in recent years, Sweden has experienced a relatively high number of such incidents [12,13]. While these accidents are not always directly related to changing weather conditions, their frequency may increase with climate change. Construction workers are thus a particularly vulnerable occupational category, and weather conditions related to climate change contribute to this vulnerability [5]. However, deregulation, pressured construction timelines, and increased construction stress are not only identified as threats to workplace safety, but also to ongoing efforts within workplaces to mitigate the effects of climate change [9,14]. This paper explores how various forms of vulnerability are constructed in construction workers’ narratives about manual labour in the construction sector, and how these vulnerabilities relate to climate change and extreme weather.

### Previous research

Previous research demonstrates that prolonged and frequent heatwaves, driven by climate change, represent one of the most serious threats to both occupational environments and public health. These extreme weather events are cited as a leading global cause of workplace fatalities and overall mortality [15,16]. A wide range of occupational groups are considered vulnerable to the impacts of climate change [17]. For instance, the increasing incidence of forest fires has led to a rise in workplace accidents among firefighters [18]. Similarly, the growing intensity and frequency of hurricanes has heightened the risks faced by rescue workers [19], as well as those involved in post-disaster clean-up operations [20]. Extended periods of drought contribute to worsening air pollution, which poses health risks to numerous occupational groups [21]. Moreover, it is argued that climate change affects all forms of work to some extent, as it fundamentally alters the conditions and experiences of working life.

All labour is made insecure by climate change given it threatens human and ecological life; impacts the capacity of capitalism to maintain stable accumulation; and often negatively impacts the capacity to labour. In other words, climate change processes – including climatic heat rises – impact the broader processes of social reproduction, work life longevity and job security, and have consequences for all workers [22] (p560).

Although workers in general are affected by climate change, the degree of vulnerability appears to vary across different groups. Studies conducted in countries such as China, the United States, and Australia identify a range of health issues associated with working in extreme heat. Among other findings, these studies suggest that older workers may be more susceptible to heat-related injuries than younger counterparts [23,24]. Some studies suggest that women are disproportionately affected by climate change [25,26], particularly in developing countries where populations are often reliant on natural resources increasingly threatened by environmental changes [27]. In contrast, other studies indicate that men may face greater challenges in certain contexts, such as prolonged exposure to extreme heat, which may heighten their risk of heat-related health issues [28]. In addition to gendered vulnerabilities, individuals who are socially, culturally, economically, or politically marginalised are considered especially at risk, as they often lack access to protective resources and adaptive capacity [29,30].

Climate change is thus viewed as a factor that amplifies workers’ vulnerability and the risks associated with their labour, but it is also understood as potentially generating resistance. As stated by Newman and Humphrys, climate change is ‘a component of precariousness in the labour process and is subject to both intensification and resistance’ [22] (p568). What is said to exacerbate workers’ vulnerability is that climate change interacts with other structural societal changes occurring in many parts of the world, such as globalisation and neoliberal policies advocating a more flexible and deregulated labour market. These developments have adversely affected many occupational groups, especially those in temporary employment [31–33].

Studies focusing specifically on the construction industry reveal that it is among the sectors most vulnerable to climate change, as it is significantly affected by extreme weather conditions such as heatwaves [5,6]. A study from the United States shows that although construction workers represent just 6% of the total workforce, they account for 36% of all occupational heat-related fatalities between 1992 and 2016 [7]. Climate change and severe weather conditions pose significant risks in the daily work carried out on construction sites, for example, strong winds can destabilise workers [34]. A literature review examining factors that influence thermal stress resilience among outdoor workers, identifies several different risk factors: individual-related heat-exposure risk factors (e.g., dehydration); environmental-related heat-exposure risk factors (e.g., high humidity), and occupational-related heat-exposure risk factors (e.g., ‘inadequate cool housing designs for rest’ and ‘the absence of occupational heat stress guidelines and adaptation strategies’) [34] (p4–5). In addition to the safety challenges and increased health risks associated with severe weather, productivity also declines [ 35–37].

Despite the wealth of available knowledge, many employees remain unaware of the risks associated with heat exposure. Several studies underscore the importance of raising awareness and implementing targeted education and training programmes to address heat-related health and safety concerns [38]. Globally, there are significant differences between countries in the measures taken to address the health challenges that climate change poses for outdoor workers. While more developed countries have introduced various initiatives, similar measures are often absent in developing countries, such as Nigeria [39]. However, it has been noted that even Sweden is slow to adapt its labour market to meet climate-related challenges [15].

Studies examining the impact of climate change on professionals also address adaptation and coping strategies—the ways in which individuals manage the effects of climate change, such as extreme heat [28]. A range of protective strategies are highlighted, including work rotation, reduced physical effort, mechanisation, and automation [34,40]. Labour adaptation is often invisible, as the work involved tends to be unpaid or underpaid [41], which emphasises the importance of recognising everyday climate adaptation, and cautions against viewing such practices merely as short-term coping strategies, since they can be highly significant for both work and the broader work environment [42]. Crucially, climate adaptation must be understood as embedded in everyday practices rather than solely in formal workplace strategies [43]. However, adaptation strategies—although developed with good intentions—can produce unintended consequences, including negative climate and environmental impacts [44].

Research shows that the prior knowledge required to develop effective strategies for coping with extreme heat depends on factors such as education, age, culture, local security systems, and national context [45]. For instance, older workers in developing countries often have lower awareness of climate change and climate adaptation compared to their counterparts in more developed nations [46].

The research field examining the vulnerability of outdoor workers in relation to climate change is extensive, but it tends to concentrate on the health consequences of extreme heat and often focuses on conditions in warmer southern countries. This exploratory study, however, foregrounds workers’ experiences, aiming to investigate the concrete practices and emotions associated with climate change, as well as how these are given meaning. It centres on Sweden—a country commonly associated with snow and cold, yet increasingly affected by the impacts of climate change. The study considers a range of extreme weather conditions, including high temperatures, strong winds, increased precipitation, and cold. It makes a significant contribution to research on the vulnerability of outdoor workers by identifying various forms of vulnerability and examining how these are shaped and intensified by extreme weather events. A key aspect of the article’s novelty and scope lies in its discussion of how the effects of climate change on construction workers acquire specific meaning and significance through the production of desynchronised temporalities. This theoretical perspective calls for a fundamental reconceptualisation of labour—not as a passive commodity simply mobilised for capital accumulation, but as embodied social subjects whose lived experiences are deeply embedded within the fabric of production. Labour, in this view, is not external to economic processes but actively shapes and is shaped by them. Challenging the abstraction of labour in neoclassical economics and aligning with more critical traditions, this perspective includes the often-invisible dimensions of social reproduction, as well as the everyday struggles and negotiations through which workers assert agency, resist exploitation, and navigate precarious conditions [47].

### Aim

This paper draws on construction workers’ narratives about their experiences of manual labour in the construction sector. The aim is to explore the various forms of vulnerability that they expressed and how they are related to challenging weather conditions and climate change. Key to this exploration is the concept of desynchronisation [48], which in this study denotes the tension between the differing temporalities that characterise work in the construction sector.

## Materials and methods

This study draws on qualitative interviews conducted with Swedish construction workers. The participants were recruited with the assistance of Byggnads, the trade union representing construction workers in Sweden. Byggnads facilitated the recruitment process by distributing an open call for participation to its members. Sixteen individuals responded to the call for participation. Two of the participants were women and 14 men, a distribution that mirrors the gender composition of the Swedish construction industry more broadly—for instance, in 2022, 98% of all carpenters in Sweden were men [49]. This number of participants was considered sufficient to achieve thematic saturation, ensuring that the data collected provided adequate depth and diversity for a robust thematic analysis. The participants represented a range of age groups and professional roles within the Swedish construction sector, and were geographically dispersed across the country. For this reason, digital interviews were chosen. BN and JL—both university lecturers holding PhDs—conducted the interviews digitally via Zoom or Microsoft Teams between March 21 and June 27 2024. Each interview lasted between 30 and 90 minutes. Ethical approval was granted by the Swedish Ethical Review Authority (Approval No. 2023-01215-01). Written informed consent was obtained from all participants, as approved by the ethics committee. The participants were informed about the study and written consent was obtained.

The advantages of digital interviews are that they can be carried out without physical meetings and, like telephone interviews, they create a safe environment for the participants [50]. However, disadvantages may include the fact that it is difficult for the interviewer to read body language, as the image on the screen provides a limited overview. A semi-structured questionnaire consisting of both specific and open questions was used as a basis for the interviews. Questions were asked about, among other things, the characteristics of the participants’ work, its pros and cons, the meanings they attributed to climate change and various weather conditions, and the concrete practices and feelings that different weather conditions give rise to. The participants were also given the opportunity to introduce their own conversation topics. All interviews were transcribed verbatim, and the quotes were translated into English and edited to improve readability.

The analytical approach adopted in this study is informed by Braun and Clarke’s framework for thematic analysis and is situated within a constructivist epistemological stance [51]. This perspective acknowledges that meaning is not discovered but constructed through interaction with the data, and is further shaped by the researcher’s interpretive engagement. The analysis unfolded through a series of interconnected phases involving all three authors: initial familiarisation with the data, generation of initial codes, identification and review of themes, definition and naming of themes, and the final production of the report.

The initial phase—familiarisation—involved an immersive and iterative reading of the material, accompanied by reflective note-taking. This process was not merely preparatory but integral to the co-construction of meaning, as early impressions and questions informed the development of codes and themes in subsequent stages. The phase of creating initial codes involved identifying aspects of the material that appeared to be relevant to the aim of the study and its research questions. ‘Searching for themes’ consisted of sorting the codes into more general themes, while ‘reviewing themes’ involved testing their viability and relevance. The phase of ‘defining and naming themes’ involved identifying both the essence of what each theme includes and ‘the “story” that each theme tells’ [51] (p92). The analytical phase ended with ‘producing the report.’

The concept of articulation served as a key analytical entry point for exploring how meaning is constructed at the micro level within the interview narratives. While the term originates in discourse theory, particularly in the work of Laclau and Mouffe [52], our use of it is more pragmatic than theoretical. Articulation is employed to trace how meaning emerges through the linking of words, ideas, and phenomena within the data. For instance, the articulation of ‘roofing’ with ‘strong winds’ and ‘safety equipment’ suggests the presence and production of a discourse of risk. In this way, articulation functions as a tool for identifying meaningful connections that inform the development of codes and themes. This approach is consistent with a constructivist epistemology, which views meaning as contextually produced and shaped through interpretive processes. To enhance reliability and mitigate the potential limitation of recall bias, we triangulated themes among the authors, one of whom did not participate in the interviews, and also encouraged respondents to provide specific, concrete examples rather than general or abstract descriptions.

During the initial inductive review of the material—referred to as the phase of familiarisation—it became apparent that the participants were critical of their working conditions and the organisation of their work. This criticism also pointed to what was later identified as an overarching theme: the narratives conveyed experiences of vulnerability. This sense of vulnerability was expressed through the ways in which the participants described the difficulties, risks, and dangers inherent in their daily tasks.

Within the interview narratives, it was possible to identify codes such as powerlessness, loss of control, subordination, uncertainty, lack of autonomy, and inadequate material resources. These codes were subsequently grouped into broader thematic categories: ‘bodily vulnerability’, ‘social vulnerability’, ‘hierarchical vulnerability’, ‘material vulnerability’, ‘market-driven vulnerability’, and ‘weather-related vulnerability’. The latter category includes sub-themes linked to specific weather conditions, such as strong winds.

### Theoretical perspectives

The material was approached from a semi-realistic perspective, meaning that the interview narratives were analysed not only as subjective expressions of personal experience, but also as accounts that shape individuals’ understanding of themselves and their surroundings, as well as their sense of identity, community, and belonging [53]. According to this view, narratives are understood as partly reflecting structural factors, including the conditions that influence how they are formed and articulated. This does not imply that the interview narratives are seen as unmediated or entirely factual representations of work in the construction sector, nor as direct reflections of the participants’ objective working conditions. Rather, they are viewed as offering meaningful insights into perceived vulnerabilities and the structural conditions that shape workers’ experiences. When multiple accounts converge, they may reveal patterns that point to broader dynamics within the sector, thereby contributing to a more nuanced understanding of labour precarity.

‘Vulnerability’, as used in this study, refers to how the participants portrayed themselves as subordinate, affected by, and exposed to their working conditions, including their perception of an increase in extreme weather events. While commonly associated with notions of victimhood, recent theoretical developments highlight the dual nature of vulnerability, emphasising that it can also serve as a source of agency and mobilisation [54,55]. This article adopts an experience-based perspective on vulnerability, focusing on identifying the moments described by construction workers as producing vulnerability. In practice, vulnerability is understood both as an outcome of working conditions and as a factor that actively shapes those conditions. Thus, ‘vulnerability’ is not treated as a fixed or unambiguous subject position, but rather as a power-laden relation to the contexts that affect individuals [55]. The concept draws attention to how work is currently organised and what this organisation means for employees, both in terms of their lived experiences and the narratives they construct around their work [56].

While vulnerability, like precarity, has been described as ‘inherent to all labor–capital relationships, to varying degrees’ [57] (p449), our analysis focuses on how vulnerability is narratively constructed within the interview material. Attention is given to how this emerges through the narratives shared by construction workers, and to what these articulations reveal about their lived experiences and structural positioning. In this sense, vulnerability is not treated as a fixed condition, but as a dynamic and contextually produced phenomenon that contributes to our understanding of labour under shifting economic and environmental conditions. In line with this, vulnerability also emerges as a position—one that individuals may align with or distance themselves from. This positioning is shaped through narrative practices and is contingent on the specific forms of vulnerability articulated in the material. Rather than being a static attribute, vulnerability is thus understood as a relational and discursively constructed stance, embedded in the ways construction workers make sense of their experiences.

Across the interviews, vulnerability was consistently linked to notions of risk, particularly in relation to perceived dangers, threats, and the potential for workplace accidents. In this context, risk is not treated as an objective or fixed phenomenon, but rather as a socially constructed framework through which various problems, hazards, and threats are interpreted and managed [58]. It is understood as a subjective experience, shaped by its relation to external conditions and mediated by broader social and discursive contexts. Consequently, the interview narratives, viewed as meaning-making processes, are informed not only by participants’ lived experiences but also by dominant discourses on climate and weather, as reproduced in workplace settings, media representations, and societal debates more broadly.

It should be emphasised that the participants did not passively accept their working conditions; rather, they demonstrated agency throughout the interviews. Their critical accounts were formed in opposition to both employers and the more general capitalist system of production that they regarded as prioritising profits over the well-being of workers. This oppositional tendency was interpreted in terms of oppositional narratives, or ‘counter stories’ [59], that find strength in feelings of vulnerability [55]. Counter stories are characterised by having an evaluative content and by being explicitly or implicitly directed towards something or someone. Oppositional narratives often reflect power relations and may involve protests or challenges to dominant societal discourses [60,61]. In this way, they function to delegitimise, challenge and potentially even to reject dominant interpretations of social phenomena.

In the efforts to contextualise the participants’ narratives, the concept of temporality serves as a guiding framework. According to Rosa, (late) modern society is characterised by multiple forms of acceleration: technological acceleration (e.g., goal-oriented developments such as faster modes of transportation), social acceleration (e.g., reduced family stability and more frequent job changes), and acceleration of the pace of life (e.g., the pervasive experience of time scarcity) [48]. Acceleration occurs both at the individual and societal levels and at different rates, which means that people’s lived experiences may become misaligned with dominant societal rhythms or temporal expectations. The concept of desynchronisation provides a valuable analytical lens for interpreting participants’ narratives, particularly regarding their struggles to make sense of conflicting temporal experiences.

As demonstrated below, the participants’ narratives reveal that their workplaces were shaped by overlapping and desynchronised temporal structures, which played a significant role in producing and exacerbating workers’ vulnerabilities closely tied to weather-related conditions.

### Findings: Weather-related vulnerability

The interview stories reflect an ambivalent approach to work in the construction sector. On the one hand, the work was discussed in positive terms and described as rewarding, varied, and instructive. The participants emphasised that they were often faced with new challenges in a positive sense. The opportunity to meet new people, make new contacts, and create networks was seen as very fulfilling: ‘You meet a hell of a lot of fun people’ (Participant 12). According to the interview stories, the participants appreciated that every day they could see the concrete results of their work, that they benefited from their professional skills during their free time, and that they felt that they were contributing to society.

On the other hand, work was also linked to ideas of a substandard work environment, characterised by tasks that were both physically and mentally demanding. Risks, including dangers, threats, and uncertainties form a central part of the narratives and contribute to the recreation of the construction worker as a vulnerable figure, and of workers as exposed to a variety of dangers and risks.

In their narratives, participants consistently framed weather and climate as central to their work, describing how increasingly frequent episodes of challenging weather complicate daily tasks. They often pointed to practical issues, including the greater number of abrupt temperature swings that can, for example, create hazards or delays with scaffolding. Difficult weather conditions also mean that the perceived risks of work increase. The research participants mentioned both direct risks—such as strong winds that can cause loose objects to blow around and injure workers—and indirect risks—such as high temperatures making workers dizzy and leading them to make mistakes. The focus on dangers and risks, and on the difficult situation this created for everyday construction work, meant that the narratives produced both positions and situations of vulnerability. The following sections outline five themes that emerged from the construction workers’ narratives about vulnerability in the workplace and how these are affected by adverse weather conditions. While the themes are interrelated, they are presented separately for the sake of clarity.

### Extreme weather and bodily vulnerability

One recurring theme in the stories about construction work in general was the vulnerability of the physical body. ‘You probably don’t leave the construction industry without having pain. You probably don’t’ (Participant 5), one participant stated, describing pain as almost inherent to the occupation. Problems with knees, shoulders, necks, and backs, as well as permanent damage from vibration and injuries caused by heavy lifting and poor ergonomics, were described as common, as was exposure to dust, chemical products, asbestos, and quartz. Everyday construction work was described as a situation of constant risk, including workplace accidents, some of which are fatal:

… as soon as you go out on the construction site, there’s danger. It could be a fall from a height. It could be cuts. Wear, noise, falling objects, so there are endless risks. A lot of black dust on construction sites these days. (Participant 2)

The interview stories were filled with descriptions of the physical body’s vulnerability in construction work, but also with detailed accounts of the factors that heightened this vulnerability: the weather. Participants recounted experiences where high temperatures are linked to fatigue, exhaustion, and confusion. Heat was said to cause personnel to act unwisely and irrationally, adding to the situation of risk. A consequence of this, the participants said, is not only that the dangers of the work increase, but also that the work takes longer to perform. Participants gave concrete examples of their adaptation strategies during heatwaves, including taking more breaks, drinking more often, and planning the work more carefully (Participant 2). Thus, the applied adaptation strategies in the form of ‘personal protective factors’ [40] result in an undesirable delay. However, and paradoxically, another consequence of high temperatures is that work takes longer, and due to time pressure, workers do not have time to take the breaks that they need to be able to perform the work safely. A carpenter described a work task in a factory during a heatwave:

We were all completely broken down. The company bought rehydration drinks and stuff like that, that we could slurp down during the day. But you still don’t get the fluids you need and there’s nowhere to cool off because there’s no air conditioning in the barracks or in the establishments where you sit and have lunch. So, you can never cool off anywhere. (Participant 4)

In this quote, the linking of difficult weather conditions and inadequate equipment results in an almost unbearable work situation, with dehydration causing workers to feel ‘broken down’. Others described even worse situations, with high temperatures causing heatstroke to the extent that workers required care and attention.

Heavy snow was also described as increasing workplace risks. It often meant that a significant portion of the day was spent making the workplace accessible, leading to delays and increased stress. Cold and precipitation were primarily associated with the risks that came with not being able to, or not having time to shovel snow, or with situations where changes in temperature made the construction site become ice-covered: ‘… it’s slipping accidents that are the biggest culprit when the cold comes’ (Participant 5). If there is snow on a roof, said one of the participants, it can be difficult to determine where it is safe to go:

Maybe when you arrive at work after a weekend, it’s snowed. Then you don’t really remember where there’s metal and where there isn’t, and then it’s easy to step on the metal and then off you go. It’s like walking on ice. (Participant 3)

Rain was described as a similar problem: ‘… scaffolding becomes slippery, ramps become slippery. […] You drop machines’ (Participant 5). According to one scaffolding fitter, a lot of rain means that the work rate decreases by 70–80% (Participant 13). One participant mentioned that rain-related delays necessitated the use of additional safety measures for work at great heights, such as harnesses and safety lines. However, the harness is considered a risk factor in itself:

...it doesn’t help much if we’re two people working on a roof and I fall and am dangling on the rope. Then the other person can’t save me, he just can’t do it, and then he has to get help. And in this harness that we have today, you can’t hang on to them for very long before you faint, and you can get really serious injuries. (Participant 3)

This participant further explained that, according to the company’s policy, when working with a harness and rope, a rescue plan should be implemented in less than five minutes. However, this is rarely possible, because it depends on where the accident occurs and whether anyone else knows that it has occurred. Thus, the applied adaptation strategies were usually in the form of ‘personal protective factors’ [40] that focused on the individual worker. Therefore, these strategies also result in an undesirable delay and ultimately tend to pit workers’ health and safety in opposition to the companies’ time planning.

### Extreme weather and material vulnerability

Work in the construction sector is largely manual and dependent upon the availability of good-quality equipment and materials. Material vulnerability captures the risks associated with inadequate equipment and materials. According to the participants, access to good-quality equipment varies with the size and character of the companies. They stated that larger companies rent equipment, which means that workers have access to the latest and most efficient equipment. Smaller companies, on the other hand, often buy tools and machines that will be used for a longer period, which can be a disadvantage because they quickly become obsolete. Older tools, the participants pointed out, are often based on internal-combustion engines; they vibrate more and produce more emissions than new electric ones, and they are heavier to work with, making the work become more time-consuming. But new equipment is expensive and participants often described situations where they felt trapped with older tools because entrepreneurs were eager to keep expenses down.

Certain technologies, and certain equipment, were thus associated with ideas of something old-fashioned and outdated, while new technologies and new equipment were understood as advantageous in terms of both usability and efficiency. In addition to the fact that the older tools were associated with various risks, such as vibration injuries, they were also said to be rooted in a different time (‘bought 20 years ago’) and temporality (time-consuming, heavy to work with), which made it difficult—and dangerous—to work at the pace expected. According to one plumber, it is the pursuit for profit that prevents entrepreneurs from investing in modern, safer machines (Participant 4).

Another question on materiality concerned the clothing that companies offer their employees, which was also seen as crucial for how work is experienced:

… it’s about what clothes you get from the company. […] What kind of selection is there? Who’s developed the selection? Is it in Stockholm or is it up in Luleå? Are they customised? No, they’re not that either. Are you allowed to try on different sizes? No, many times, it’s the managers who bring a bag here: ‘You should have this.’ And it’s also, what gloves are you allowed to wear? Are you allowed to wear the best winter gloves? No, you can’t, because they’re too expensive. […] So many construction workers actually have to pay for this [inadequate clothing] with their bodies. (Participant 4)

When discussing vulnerabilities linked to material conditions, participants frequently emphasised the impact of weather-related factors. They described a range of practical challenges arising from extreme temperatures, for instance, battery-powered tools tend to lose power in cold weather and are prone to overheating in high heat. These environmental conditions were seen as directly affecting both the efficiency and safety of their work.

Weather-related problems with equipment were also considered to lead to reduced work efficiency, leading to various work phases taking longer than usual, which was experienced as both frustrating and stressful. The participants also pointed out that just as with high temperatures, snow and rain as well as the increasingly strong winds result in more time-consuming work: ‘Tasks that would normally take an hour can take a whole day’ (Participant 14). For example, tarpaulins blow apart more often than before, and scaffolding and sheets must be anchored more carefully. Due to time pressure, workers reported having been forced to weld in the rain, which they described as very dangerous, because it can lead to electric shocks (Participant 14).

The interview stories illustrate that not only extreme weather but also temperature *fluctuations* can lead to an increased need for work to be covered when a cold morning turns into a warm afternoon. Precipitation such as heavy rainfall or snow can make it difficult to protect all insulation, and instead it is transported in batches, which means more work and extended working hours, and, paradoxically, more fossil-fuel emissions (Participant 4).

Clothing was also described as a problem that increased with extreme weather. Participants mentioned that employers often prioritise cheap workwear instead of clothing that is best suited to the prevailing weather and climate conditions, which contributed to an increase in material vulnerability during difficult weather. They also pointed out that company policies do not permit workers to wear shorts, which is understandable from a safety point of view, but also makes the work more arduous at high temperatures. The fact that many employees are required to wear so-called high-visibility clothing was also seen as a problem in hot weather. These clothes were likened to plastic bags that trap all moisture and only make it hotter. While the participants generally respected and supported safety policies, extreme weather evoked feelings of being trapped between safety and well-being, sometimes leading them to ignore safety regulations.

### Extreme weather and hierarchical vulnerability

Although the participants had experience working in companies of varying sizes, their narratives converged around a shared sense of vulnerability. This vulnerability was closely tied to their positioning within rigid organisational hierarchies—particularly in larger companies—where clear and impermeable divisions existed between different groups, most notably between top management and blue-collar workers. These structural separations contributed to a sense of exclusion and powerlessness that was consistently reflected across the interviews:

We have people who provide information at construction starts and things like that. But it’s nothing like... well, you really don’t know what they’re doing, but they come out and provide information about things like that. But it’s kind of above our pay grade you could say. (Participant 5)

The information coming from management was described as generally having a one-way character, with construction workers not being part of the work planning, which takes place at a higher level within the organisation.

Since extreme weather often prolongs construction times and increases costs, suggestions to adapt to the weather by making purchases were sometimes met with explicit references to hierarchy that were not perceived as possible to challenge:

There are entrepreneurs who say things like: ‘Sit down and shut up, do as I say. You get this, you should use this stuff.’ Dinosaur stuff that he bought when he started the company 20 years ago that’s terrible to use compared to modern stuff. (Participant 4)

…if the project isn’t going to be financially viable, it becomes very clear that they [the managers] think it’s *their* money being wasted or that ‘it’s *my* money that’s being spent on this now when I have to compensate your [lack of] effort by contributing 20% myself.’ (Participant 8)

In both quotes above, vulnerability is present because the hierarchical relations are made *personal,* and employees were personally blamed if a project did not seem to be on time due to weather. Because workers generally accepted the hierarchies—presenting themselves as subordinates to managers, supervisors, to the companies more generally, as well as to an overarching economic system—they sometimes refrained from offering criticism or making demands.

Overall, hierarchical vulnerability can be understood as an expression of class relations. The narratives construct a classed position, closely associated with manual, blue-collar labour, as inherently vulnerable. This vulnerability was frequently articulated as a key reason for workers’ limited ability to influence their working conditions, particularly during episodes of extreme weather. In this sense, class position is not only structurally imposed but also narratively produced, shaping how agency and constraint are experienced and expressed.

### Extreme weather and social vulnerability

Some participants worked in service departments, travelling between several different assignments, sometimes during the same day, including to private customers. Social vulnerability concerns the relationships with customers, particularly workers being vulnerable to their discontent when weather requires special measures or leads to delays. The following story is about working in high temperatures:

It gets a little strange, if I bring—let’s say to your living room where I’m going to change the radiator—bring a fan with me to cool me down. Then you might think: ‘how much power does it consume?’ Yes, I can promise you that, it can happen, it has happened with fans: ‘how much power does it consume, should I pay for it or not?’... People look at the clock a lot and everything around them. (Participant 1)

This story illustrates a socially stressful situation, as customers sometimes object when workers bring equipment that uses electricity, which incurs additional costs but is not strictly necessary for the work itself. In another example, which involved roofing during a heatwave, the roofers were scolded by a customer who thought the work was taking too long (Participant 4). This customer’s reaction led to a socially awkward and difficult situation:

I’ve been on jobs when it’s been extremely hot, when we’ve put a roof on a customer’s house, for example, and got a lot of scolding because we were far too slow. But it was just that you couldn’t walk on the roofing felt because it melted, and you tore it apart with your foot when you just stepped on the roof slope. It definitely becomes a problem during certain periods and days. (Participant 4)

This example describes work being delayed due to hot weather, which also meant a difficult social situation due to the customer’s reaction. Another social risk reflected in the material concerns leisure time. When taking about working in difficult weather conditions, especially high temperatures, the participants recalled situations in which they had become completely exhausted and could not cope with a ‘normal’ family life after work, which, they asserted, could affect both their life partners and children.

### Extreme weather and market-driven vulnerability

… money rules in some way, that’s how it is... (Participant 15)

Everyday work was repeatedly articulated with change, especially changing structural conditions within the labour market. Market-driven vulnerability describes how a situation of vulnerability was ascribed to global processes of neoliberalism and marketisation, characterised by deregulation, privatisation, and competition. In the interviews, such processes were associated with a pervasive economisation of the construction industry, including increasingly shorter build-times, increased involvement of subcontractors and project-based constructions, increased reliance on global migrant labour who speak neither Swedish nor English, and a fragmentation of work and a subsequent loss of control, sense of cohesion, and professional pride. There was a strong association between such processes and an elevated risk of accidents. As a consequence, the participants said, individual workers tend to take personal responsibility for delays, feel increasingly stressed and pressured to work harder and faster, and consequently feel compelled to compromise workplace safety. In their stories about the situation, the participants made comparisons over time, with past periods being attributed particular advantages:

Yes, it gets worse and worse with each passing year. Go back 15–20 years and it wasn’t like this at all. Back then, there was more safety awareness […] Now we should just make as much money as possible, build as cheaply as possible, and charge as much as possible. (Participant 6).

Market-driven vulnerability was thus re-created when the participants attributed the causes of a transformed work situation to external factors on a global scale and beyond their control. Just as with the other types of vulnerability, market-driven vulnerability tended to be severely affected by the weather, or rather, it tended to result in reduced preparedness to deal with extreme or unpredictable weather. This was primarily because stress, reduced opportunities to take important breaks for rest and hydration, language barriers that caused confusion, and the reduced cohesion and solidarity resulting from increased staff turnover led to greater carelessness with safety and an increase in misunderstandings and miscommunication.

## Discussion: Extreme weather, vulnerability and temporal tensions in manual labour

Research increasingly demonstrates that climate change exacerbates vulnerabilities among outdoor manual labourers [17]. Although Sweden’s climate differs from hotter regions like India [62] or the southern U.S. [7], Swedish construction workers nonetheless report significant impacts from extreme weather, including heatwaves, heavy rainfall, and strong winds. While direct causal relationships between climate change and workers’ experiences cannot be assumed, these weather conditions, which are expected to intensify with climate change [63], appear to amplify existing risks and insecurities in the workplace. This study foregrounds construction workers’ recounted experiences of extreme weather. It shows the costs borne by workers as they manoeuvre a range of five different vulnerabilities, and reveals that vulnerability manifests across multiple dimensions: organisational, structural, material, social, and physiological. Organisationally, workers face hierarchical subordination, while structurally, they experience a loss of control due to shifting work conditions. Material vulnerabilities include inadequate equipment and clothing, which vary depending on employer resources. Socially, workers report increased stress and reduced cohesion and solidarity [64], especially in contexts with many self-employed workers without an employment contract [65].

What became clear during interviews, was how extreme weather—the problems, dangers, and risks that stem from working in high temperatures, heavy rainfall, and/or strong winds [34]—tended to amplify the risks and feelings of vulnerability that were already associated with everyday construction work in a deregulated, neoliberal labour market. Extreme weather compounds these challenges, increasing stress, reducing productivity, and making work more hazardous [24,66,67]. Participants described how weather-related delays sometimes led to blame being placed on workers, further intensifying their sense of powerlessness. One aspect that seemed particularly significant was the way in which extreme weather conditions tended to heighten workers’ feelings of vulnerability by causing them to lose control over their work situation [68] and by reducing working capacity [36,37,69] as work becomes more time-consuming when new tasks are added, pace must be reduced, and more pauses are needed.

In the workers’ accounts of their everyday work-life experiences, and the difficult conditions and risks they identified as part of these, temporal dynamics consistently appeared as a key explanatory dimension. A key theme emerging from the interviews is the feeling of temporal dissonance, characterised by conflicting perceptions and structures of time within the workplace, which contributed to increased experiences of vulnerability. Three distinct temporalities were identified—ideal temporality, everyday work temporality, and extreme weather temporality—each affecting the various forms of weather-related vulnerability described by the participants (bodily, hierarchical, material, social, and market-driven).

‘Ideal temporality’ refers to the employer-imposed temporal regime structured around the imperatives of capitalist production. It privileges speed, efficiency, and adherence to strict deadlines, often at the expense of the lived realities and constraints of workers on the ground. This temporal logic is not merely a scheduling preference but a disciplinary mechanism that enforces productivity norms and marginalises alternative rhythms of work. In the narratives of construction workers, ideal temporality appeared as a dominant force—an unyielding driver of pace that was perceived as non-negotiable. Any attempt to challenge or renegotiate this temporal framework was interpreted not as a legitimate concern, but as a delay or obstruction to progress. This underscores the asymmetrical power relations embedded in temporal control and highlights how time itself becomes both a driver of vulnerability and a site of struggle within labour processes.

‘Everyday work temporality’ included the time it takes to finish a certain work task, but also the time it takes when important things need to be communicated with someone whose language you do not speak. Rooted in workers’ lived experiences, this temporality reflects the actual time required to complete tasks. It was perceived as complex and as having inherent limits to speed, making it resistant to acceleration [48]. Everyday work temporality was also not without its disturbances [70], including delays caused by communication barriers, safety concerns, and routine disruptions. It often conflicted with ideal temporality, leading to increased vulnerability through stress and perceived underperformance. As shown by Newman and Humphrys in their study of heat stress among construction workers, many characterised their own attempts to protect their health and safety in high heat conditions as ‘fundamentally incompatible with their employers and managers, who were necessarily focused on maintaining a particular intensity of labour in order to meet deadlines and complete jobs’ [22] (p567).

‘Extreme weather temporality’ arises in response to adverse weather conditions, imposing constraints on work speed and requiring additional safety protocols and rest periods to mitigate risk. These adaptations are rarely accounted for in project planning, making them appear as optional or even obstructive. Consequently, workers may forgo essential precautions in an attempt to mitigate the vulnerability inherent in being blamed for delays, thereby increasing their exposure to risk.

The differing rates of acceleration among these temporalities create incompatibilities that lead to a state of desynchronisation [48]. The economically driven ideal temporality dominates, framing any deviation as a failure. This hierarchy pressures workers to conform to unrealistic timelines, even under dangerous conditions, reinforcing their vulnerability by putting strain on an already stressful work context [55].

While it would be possible to view less accelerating societal spheres such as the sphere of everyday work as constituting a counter-trend to modern acceleration, Rosa argues that they merely denote ‘the (retreating) limits of social acceleration; they are not counter-powers at all’ [48] (p17). However, the interviews reveal moments of agency and even resistance. Workers expressed critical views of the accelerating pace of work and the disregard for their safety. These narratives, while rooted in vulnerability, also carried a political charge [55]. They can be seen as oppositional narratives [59–61] that challenge dominant norms and potentially serve as a basis for collective action [54], at least within the realm of the interviews. Given the growing legitimacy of climate discourse in Sweden, the EU, and globally, these experiences could provide a platform for workers to advocate for safer, more humane working conditions. Climate change, while a source of risk, might also become a catalyst for labour mobilisation and policy change.

Drawing on interview data, and foregrounding the often-invisible dimensions of social reproduction alongside the everyday struggles and negotiations through which workers assert agency, resist exploitation, and navigate precarious conditions [47], this approach enables a critical illumination of the hidden costs borne by labour [70]. These costs become particularly pronounced in the context of intensifying extreme weather events, where the intersection of environmental vulnerability and rigid labour regimes exacerbates the precarity of workers’ lives and livelihoods. Such dynamics underscore a broader tension in contemporary debates on just transition centred around labour and climate justice [71–72], highlighting the discursive impact of ‘green’ and other fantasies of the future [73–74], and how the promise of equitable and climate‑resilient labour futures is frequently undercut by policy arrangements that insufficiently account for the lived realities of frontline workers [75]. In this sense, the findings speak directly to concerns that current labour‑policy frameworks risk reinforcing, rather than alleviating, inequities unless worker safety, wellbeing, and collective voice are placed at the centre of climate adaptation strategies. In such settings, the ideal of a just transition that equitably addresses the social dimensions of climate adaptation and mitigation remains largely aspirational. By making these dynamics visible, the analysis challenges dominant narratives that obscure the socio-environmental burdens disproportionately shouldered by labouring bodies.

### Limitations

While the study design offers valuable insights, some methodological limitations should be acknowledged. First, the findings rely on self‑reported data, capturing subjective perceptions rather than objectively measured exposures or risks. Although qualitative research does not aim for statistical representativeness, the views captured here may not fully encompass the range of experiences present in broader or more diverse groups. Second, the reliance on semi‑structured interviews introduces potential social desirability and recall bias; participants may have framed experiences in ways consistent with workplace norms or with what they believed interviewers’ expectations to be, and retrospective accounts of weather‑related challenges may also reflect imperfect memory. To mitigate these potential biases, we encouraged respondents to provide specific, concrete examples rather than general or abstract descriptions. Third, the gender imbalance in the sample—two women among sixteen participants—limits the ability to explore gender‑specific experiences and may underrepresent women’s perspectives. Fourth, the study’s Swedish context may restrict transferability, as labour conditions, regulatory frameworks, and climate‑related risks vary across countries. Fifth, the involvement of two researchers in both data collection and analysis provided rich insights through direct engagement and exploration of emerging themes, although it may also have influenced interpretation, despite the use of reflexive practices and peer debriefing to mitigate preconceptions. To enhance the trustworthiness of the analysis, interviews were conducted until thematic saturation was reached, reflective memoing was used to document analytic decisions, and themes were triangulated among the authors—one of whom had not participated in the interviews—to strengthen interpretive rigour. These limitations should be considered when interpreting the findings.

## Conclusion

In conclusion, this study offers valuable insights into how construction workers in Sweden experience and articulate the impact of adverse weather conditions on their working lives. By focusing on the micro-level of daily labour, it addresses a notable gap in existing research and demonstrates how specific weather conditions shape both work practices and perceptions. The analysis identifies multiple forms of vulnerability that arise in relation to environmental exposure, organisational structures, and classed positions within the labour hierarchy, and highlights how adverse weather intensifies temporal pressures by widening the gap between actual working time and formal project schedules. These findings underscore the importance of considering environmental, organisational, and temporal dimensions in discussions of labour vulnerability and just transitions within the construction sector.

## Supporting information

S1 File Information for research participants. (DOCX)

S2 File Information for research participants in Swedish. (DOCX)
