# Supplementary material for: Vulnerabilities, extreme weather and temporal tensions as experienced by construction workers in the Swedish construction sector
Source: PLoS One. 2026 Apr 24;21(4):e0345707. doi: 10.1371/journal.pone.0345707 (PMC13108760; doi:10.1371/journal.pone.0345707)
Supplement: S1 File — (DOCX) [file pone.0345707.s001.docx]

**Information for Research Participants**

We would like to ask whether you are willing to take part in a research project. This document provides information about the project and about what participation entails.

**What is the project about, and why am I being asked to participate?**

Climate change, with rising temperatures, increased precipitation, and stronger winds, is affecting large parts of society, not least certain vulnerable groups in working life. The purpose of the project is to investigate how healthcare personnel, construction workers, and road workers experience and manage a changing climate and altered weather conditions in their work.

You are receiving this letter because you are either employed in healthcare or work as a construction worker or road worker.

We have obtained your name through your workplace or because someone else has recommended you.

Umeå University is the principal research organisation for the project. The principal research organisation is the institution that is responsible for the project. The application has been approved by the Swedish Ethical Review Authority. The reference number for the ethical review is **Dnr 2023‑01215‑01**.

**How will the project be carried out?**

Participation in the project involves agreeing to be interviewed, either through an in‑person meeting, by telephone, or via a digital platform. You decide the location yourself. For example, it may take place at your workplace, in your home, or at Umeå University. You will be asked questions about your work, working conditions, and how you experience and manage challenging weather conditions. The interview will take approximately one hour.

**Possible consequences and risks of participating in the project**

Your participation in the project does not involve any direct risks to you. You decide which questions you wish to answer, and you may also terminate the interview at any time. Ending your participation will not affect your employment situation. However, questions about work and working conditions may potentially evoke certain emotions.

**What happens to my data?**

The project will collect and register information about you. The material from the recorded interviews will be transcribed into text. During this process, information that could identify you will be removed. After transcription, the text material will be stored in accordance with Umeå University’s regulations for the management of research data.

Your responses will be handled in such a way that unauthorized persons cannot access them.

When the project has been completed, the pseudonymised data will be archived at the Department of Culture and Media in accordance with Umeå University’s guidelines.

Umeå University is the data controller responsible for your personal data. In accordance with the EU General Data Protection Regulation (GDPR), you have the right to access, free of charge, the personal data about you that are processed within the project, and, if necessary, to have any inaccuracies corrected. You may also request that your personal data be erased and that the processing of your personal data be restricted. However, the right to erasure and restriction of processing does not apply when the data are necessary for the research in question. If you wish to access your data, please contact Bo Nilsson (see contact details below). The Data Protection Officer can be contacted at **pulo@umu.se**. If you are dissatisfied with how your personal data are being processed, you have the right to submit a complaint to the Swedish Authority for Privacy Protection (Integritetsskyddsmyndigheten), which is the supervisory authority.

**How will I receive information about the results of the project?**

If you wish to access your own responses, please contact the researcher responsible for the project. All publications resulting from the project will be available via **diva-portal.org**.

**Insurance and compensation**

No compensation will be provided.

**Voluntary participation**

Your participation is voluntary, and you may choose to withdraw from the project at any time. If you decide not to participate or to withdraw your participation, you do not need to provide a reason.

If you wish to withdraw from the project, please contact the person responsible for the project (see below).

**Person responsible for the project**

The person responsible for the project is **Bo Nilsson**, Professor of Ethnology, Umeå University, SE‑901 87 Umeå.
Email: **bo.nilsson@umu.se**
