# Supplementary material for: Vulnerabilities, extreme weather and temporal tensions as experienced by construction workers in the Swedish construction sector
Source: PLoS One. 2026 Apr 24;21(4):e0345707. doi: 10.1371/journal.pone.0345707 (PMC13108760; doi:10.1371/journal.pone.0345707)
Supplement: S2 File — (DOCX) [file pone.0345707.s002.docx]

# Information till forskningspersoner

Vi vill fråga dig om du vill delta i ett forskningsprojekt. I det här dokumentet får du information om projektet och om vad det innebär att delta.

## Vad är det för ett projekt och varför vill ni att jag ska delta?

Klimatförändringar med stigande temperaturer, ökad nederbörd och hårdare vindar påverkar stora delar av samhället, inte minst vissa utsatta grupper i arbetslivet. Syftet med projektet är att undersöka hur vårdpersonal, byggnadsarbetare och vägarbetare i sitt arbete upplever och hanterar ett förändrat klimat med förändrade väderförhållanden.

Du får detta brev därför att du antingen är anställd inom vården, är byggnads- eller vägarbetare.

Vi har fått ditt namn genom din arbetsplats eller genom att någon annan tipsat om dig.

Forskningshuvudman för projektet är Umeå universitet. Med forskningshuvudman menas den organisation som är ansvarig för projektet. Ansökan är godkänd av Etikprövningsmyndigheten, diarienummer för prövningen hos Etikprövningsmyndigheten är Dnr 2023-01215-01.

## Hur går projektet till?

Deltagande i projektet innebär att du går med på att bli intervjuad, antingen genom att vi träffas fysiskt, via telefon eller en digital plattform. Plats bestämmer du själv. Det kan t.ex. vara på din arbetsplats, i din bostad eller på Umeå universitet. Du kommer att få frågor om ditt arbete, arbetsförhållanden och om hur du upplever och hanterar försvårande väderförhållanden. Intervjun tar cirka 1 timme.

## Möjliga följder och risker med att delta i projektet

Ditt deltagande i projektet innebär inga direkta risker för dig. Du väljer själv vilka frågor du vill svara på och du kan också avbryta intervjun när som helst. Ett avbrott kommer inte att påverka din anställningssituation. Möjligtvis kan frågor om arbete och arbetsförhållanden väcka vissa känslor.

## Vad händer med mina uppgifter?

Projektet kommer att samla in och registrera information om dig. Det som framkommer under de inspelade intervjun kommer att transkriberas till text. Då raderas också information som gör att uppgifterna kan härledas till dig. Efter transkribering förvaras textmaterialet i enlighet med Umeå universitets regler för hantering av forskningsmaterial.

Dina svar kommer att behandlas så att inte obehöriga kan ta del av dem.

När projektet är avslutat kommer de pseudonymiserade uppgifterna att arkiveras vid Institutionen för kultur- och medier i enlighet med Umeå universitets riktlinjer.

Ansvarig för dina personuppgifter är Umeå universitet. Enligt EU:s dataskyddsförordning har du rätt att kostnadsfritt få ta del av de uppgifter om dig som hanteras i projektet, och vid behov få eventuella fel rättade. Du kan också begära att uppgifter om dig raderas samt att behandlingen av dina personuppgifter begränsas. Rätten till radering och till begränsning av behandling av personuppgifter gäller dock inte när uppgifterna är nödvändiga för den aktuella forskningen. Om du vill ta del av uppgifterna ska du kontakta Bo Nilsson (se kontaktuppgifter nedan). Dataskyddsombud nås på [pulo@umu.se](mailto:pulo@umu.se). Om du är missnöjd med hur dina personuppgifter behandlas har du rätt att ge in klagomål till Integritetsskyddsmyndigheten, som är tillsynsmyndighet.

## Hur får jag information om resultatet av projektet?

Om du vill ta del av dina svar kontaktar du forskningsansvarig. Alla publikationer är åtkomliga via diva-portal.org.

## Försäkring och ersättning

Ingen ersättning utgår.

## Deltagandet är frivilligt

Ditt deltagande är frivilligt och du kan när som helst välja att avbryta deltagandet. Om du väljer att inte delta eller vill avbryta ditt deltagande behöver du inte uppge varför.

Om du vill avbryta ditt deltagande ska du kontakta den ansvariga för projektet (se nedan).

## Ansvarig för projektet

Ansvarig för projektet är Bo Nilsson, professor i etnologi, Umeå universitet, 901 87 Umeå. E-post: bo.nilsson@umu.se. Telefon: 090-786 99 62.
